# A finite element model for predicting impact-induced damage to a skin simulant

**DOI:** 10.1038/s41598-024-60369-z

**Published:** 2024-06-04

**Authors:** Syed A. Imam, Angus C. Hughes, Matt Carré, Heather Driscoll, Keith Winwood, Prabhuraj Venkatraman, Tom Allen

**Affiliations:** 1 Canterbury Christ Church University, Canterbury, CT1 1QU UK; 2 Manchester Metropolitan University, Manchester, M1 5GD UK; 3 Department of Mechanical Engineering, University of Sheffield, Sheffield, S1 3JD UK; 4 Advanced Manufacturing Research Centre (AMRC), Sheffield, S60 5BL UK

**Keywords:** Mechanical engineering, Biomedical engineering

## Abstract

A finite element model was developed for assessing the efficacy of rugby body padding in reducing the risk of sustaining cuts and abrasions. The model was developed to predict the onset of damage to a soft tissue simulant from concentrated impact loading (i.e., stud impact) and compared against a corresponding experiment. The damage modelling techniques involved defining an element deletion criterion, whereby those on the surface of the surrogate were deleted if their maximum principal stress reached a predefined value. Candidate maximum principal stress values for element deletion criteria were identified independently from puncture test simulations on the soft tissue simulant. Experimental impacts with a stud were carried out at three energies (2, 4 and 6 J), at three angular orientations (0°, 15° and 30°) and compared to corresponding simulations. Suitable maximum principal stress values for element deletion criteria settings were first identified for the 4 J impact, selecting the candidates that best matched the experimental results. The same element deletion settings were then applied in simulations at 2 and 6 J and the validity of the model was further assessed (difference < 15% for the force at tear and < 30% for time to tear). The damage modelling techniques presented here could be applied to other skin simulants to assess the onset of skin injuries and the ability of padding to prevent them.

## Introduction

World Rugby™ have defined a set of regulations named the Body Padding Performance Specifications^1^. These regulations define the ergonomics, construction, sizing and design along with the performance requirement of body padding used in Rugby Union. They state that body padding should only “protect against cuts and abrasions”. Cuts are typically caused by interaction with another player or their footwear^2,3^, whereas abrasions are usually from contact with artificial turf^4–6^. With technological advances, these regulations must be “future-proof” to ensure the materials and design of rugby padding do not become detrimental to the nature of the sport and player welfare.

Testing to assess how padding influences the risk of skin injuries, particularly cuts and abrasions, could involve cadaveric human specimens, but there are limitations to this approach. These limitations include anthropometric variations^7^, degradation and damage from storage and repeated testing and ethical considerations. Artificial simulants have been developed to mimic human soft tissue^8,9^ but these also have limitations with respect to testing padding. These simulants can degrade with repeated impacts^10,11^ and a new sample is often required after the one being tested is damaged, e.g., following a test that mimics a cut or abrasion. To overcome such limitations of physical testing, Imam et al.^12^ developed a Finite Element (FE) model for simulating impact on a soft tissue simulant, using the commercial software Ansys© LS-DYNA. Developing such a model to simulate impact-induced damage to the skin simulant would allow prediction of the ability of padding to protect against such injuries.

Research applying FE models to predict damage of materials tends to focus on ballistic impacts^13,14^, armour development^15,16^ and automobile crashes^17–19^. With the objective of developing an FE model for predicting skin injuries, it is important to understand the options available to predict injury and its extent. Simulating material damage, or injury prediction, in an FE model, is possible by applying damage detection (permanent change in element shape) or element deletion (removal of the element once predefined criteria are met) criterion. For example, commercial FE software by Ansys© has some material models with built-in element failure criteria^20^ (e.g., *MAT_COMPOSITE_DAMAGE, *MAT_CONCRETE_DAMAGE, *MAT_PLASTICITY_WITH_DAMAGE) which can be used for predicting material damage under different loading conditions. With such material models, an element in the mesh is removed if the assigned failure criteria are met, simulating damage. Most of the material models with fracture prediction capabilities are either ductile (metallic), having a defined plastic phase before fracture, or brittle (such as wood or ceramic), where the fracture may occur at low strain^12,20^. Skin tissue simulants are usually elastomeric, with comparatively smaller plastic regions and large strains before fracture.

In relation to sporting goods, Fortin-Smith et al.^21^, and later Campshure et al.^22^, simulated baseball-on-bat impacts to predict the nature of wooden bat breakage, using the commercial FE software LS-DYNA (which is now part of Ansys©). Using material properties for wood and adding element deletion criteria based on a maximum principal strain threshold, they were able to simulate bat breakage following a baseball impact. The failure strain of the wood was obtained in a Charpy impact test on small samples and then applied to the corresponding material model using an element erosion card (*MAT_ADD_EROSION)^22^. This element erosion card enables element deletion criteria to be added to a material model. These element deletion criteria inform the solver to “erode” or delete an element if the criterion is met^17^. Various element deletion criteria are available in Ansys LS-DYNA^20^. Each element deletion criterion can be applied independently, and if one or more of them (depending on the user setting) are satisfied, the element is removed from the simulation.

The aim of this study was to develop and validate the FE model of the soft tissue simulant of Imam et al.^12^ to predict the onset of impact-induced damage to the outer surface of the simulant (i.e., skin damage)^12^.

## Methods

To support the development of an FE model of a stud impact on the shoulder surrogate of Imam et al.^12^, in terms of gaining experimental data for validation, the skin stamping test was adapted from the World Rugby™ Studs and Outsoles Specification^23^: Test B-Skin Stamping Test. The skin stamping test method was chosen as it provides a scenario (stud and skin contact) like those that may occur and cause a cut, during rugby. The skin stamping test specifies an impact with an energy of 4.17 J, achieved by dropping an 8.5 kg mass with a stud impactor from 50 mm (impact speed of ~ 1 m s^−1^). The regulation does not state the basis for the parameters of this skin stamping test. Oudshoorn et al.^24^ argue that the skin stamping test uses an impact mass that is higher and an impact speed and energy that are lower than those of stamping events in rugby. As such, a test with a lower mass dropped from higher was thought to be more representative of stamping events during rugby than the Skin Stamping Test. Oudshoorn et al.^24^ also noted that the skin stamping test does not account for oblique impacts.

The effective total mechanical energy of a foot strike during walking or running has been reported as 0.24–6 J^25–30^. To simulate a stud impact on the soft tissue simulant the impact test setup from Imam et al.^12,31^ was modified by replacing the flat-faced impactor with an aluminium stud (18 mm Stud Set-Carta Sport, ~ 12 g). Following this modification to the test setup, the mass of the impactor was 3.65 kg, which is closer to the effective mass (0.5–2.9 kg) reported by Oudshoorn et al.^24^ for a foot strike (during a simulated rugby stamping event) than the 8.5 kg mass stated in the Studs and Outsoles Specification: Test B-Skin Stamping Test. A 4 J impact energy was chosen to support the development of the impact test and the corresponding FE model, in terms of identifying suitable element deletion criteria settings. The 4 J impact energy is close to the energy specified for the Skin Stamping Test, as well as that for a foot strike during walking or running (0.24–6 J)^25–30^, and when a 3.65 kg mass is dropped (from 0.11 m assuming no friction in the test rig) it gives an impact speed of 1.5 m s^−1^ (~ 10% below the reported 1.6–1.7 m s^−1^ vertical foot speed at ground contact^32–34^).

The shoulder surrogate used for testing matched the design of Imam et al.^12^. This surrogate had a hemicylindrical metal core (⌀ 75 mm) covered by a 15 mm thick layer of silicone (mimicking soft tissue) which included a 1.5 mm thick outer layer of synthetic chamois leather (mimicking skin). Five silicone samples were moulded along with an outer layer of chamois leather, allowing for repeated testing. Quasistatic compression testing was undertaken on these silicone samples, both before and after impact testing, to check their consistency, as detailed in the Supplementary Material-1-Section 2. No significant differences were found when comparing samples before testing or before and after testing (*p* > 0.05: range 0.078–0.476).

Pilot testing with a fresh shoulder surrogate showed the chamois leather to tear at impact energies over 2.5 J when struck with the 3.65 kg stud impactor in the 0° orientation. This pilot testing showed only superficial damage (no tear) to the chamois leather for 2 J impacts, so only one surrogate was tested at this energy. Two samples were tested at the other energies of 4 and 6 J (for all three angular orientations). The impacts at each energy were carried out at 15° and 30° angles (between the horizontal axis and the angle vice) along with perpendicular impacts (0° between the vertical axis and impactor). These test angles covered the range of roll and pitch reported by Oudshoorn et al.^24^.

### Stud impact testing

**Figure 1:**
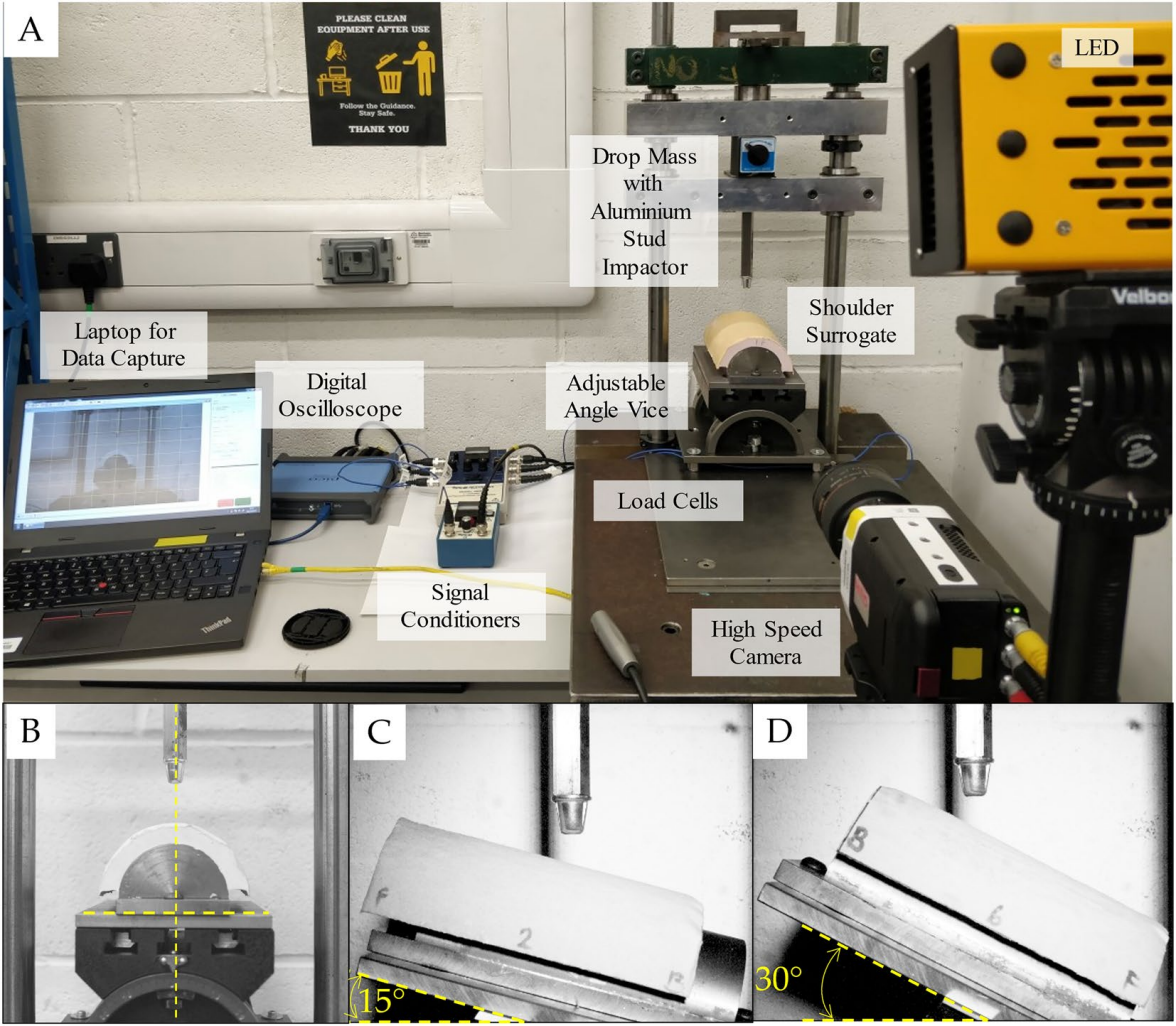
(**A**) Stud impact testing showing the silicone surrogate mounted for perpendicular impact. Impact test setup for (**B**) 0° orientation (central impact), (**C**) 15° after rotation and (**D**) 30° after rotation.

The angle vice was positioned horizontally under the stud impactor (Fig. 1A, B) for 0° impacts. For oblique impacts, the angle vice was rotated 90° around the vertical axis and then inclined to angles of 15° and 30° (Fig. 1C, D) to ensure impacts were along the longitudinal axis of the surrogate. The angle of the vice was checked with a digital inclinometer (SPI ®-Digital Inclinometers, UK; accuracy ± 0.1°). Each sample was impacted three times at the assigned energy and each angle setting (total of nine impacts), with at least one minute between impacts. The position of the silicone on the hemicylindrical core was adjusted between tests to move the impact location by at least 1 cm.

The data acquisition system, and settings, matched those of Imam et al.^12^, and are only briefly summarised here. The four load cells (208C05-Force Sensor, PCB Piezotronics) located under the base of the angle vice sampled at 10 kHz. These load cells were connected to an oscilloscope (PicoScope®, Version 6, Pico Technology) via one 3-Channel (480B21, PCB® Peizotronics) and one 1-Channel (480E09, PCB® Peizotronics) ICP® sensor signal conditioners (480B21, PCB®) to record impact force. Each impact was filmed with a high-speed camera (Phantom Miro R111, Vision Research, USA) with a zoom lens (Nikon AF Nikkor 24–85 mm 1:2.8–4 D, Nikon Corporation, Japan)^12^ and lit with light-emitting diode lights (Multiled LT-V9-15, GS Vitec, Germany).

After three impacts at a specific energy and angle, the dimensions of any damage caused to the silicone (i.e., indent, hole, tear) by the stud were measured with a vernier calliper and photographed (Nikon D3200 with zoom lens: AF-Micro Nikkor 60 mm F 1:2.8 D, Nikon Corporation®, Japan). The camera was placed on a tripod and activated with a remote trigger. The force and time values corresponding to a tear in the silicone (identified by a drop in force and compared with HSV images—for details see Online Supplementary Materials—Section 5. Experimental Stud Impact Force Trace Breakdown and Results) for each test orientation were compared.

### Skin simulant puncture test

**Figure 2:**
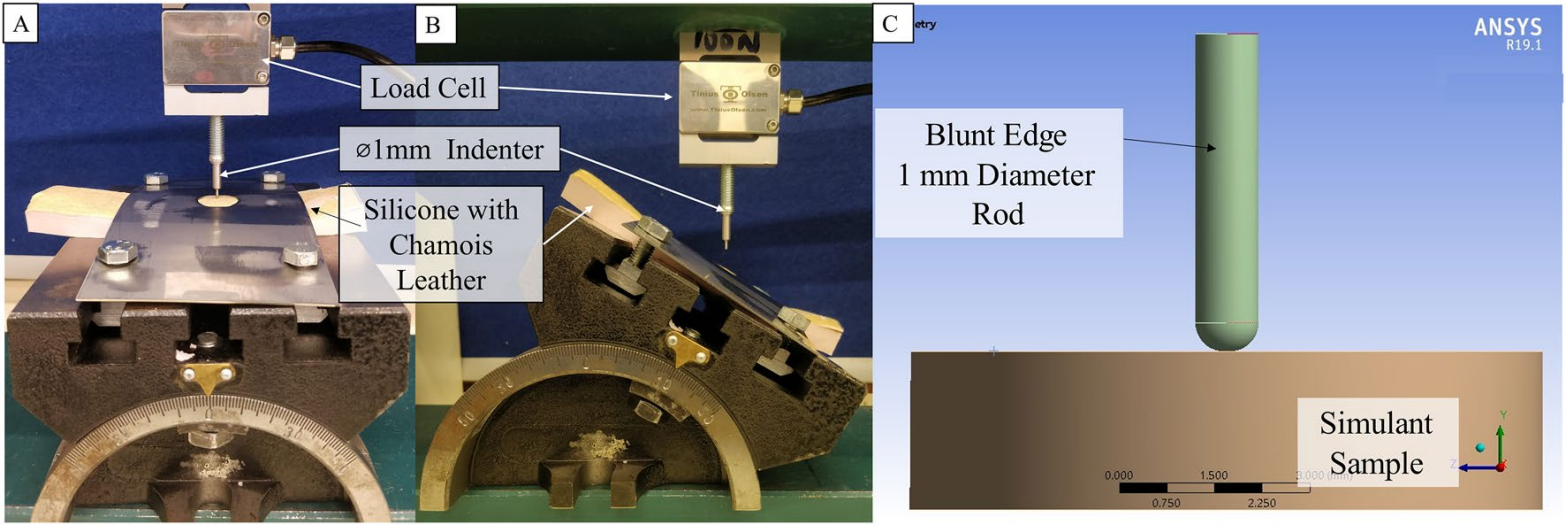
Skin puncture Test Setup at (**A**) flat and (**B**) 30° orientation along with the (**C**) CAD setup of the puncture test simulation.

A SynDaver™ (Florida, USA) skin tissue simulant (Caucasian with 2 N Puncture rating) was used initially on an early shoulder surrogate prototype and then replaced by silicone and chamois leather (IC200, Kent Car Care, Manchester, UK) (due to lower cost)^12^. SynDaver™ state that their skin tissue simulants have a puncture rating of either 2, 4, 6 or 10 N. The puncture rating of the chamois leather used on the surrogate design impact tested here was measured to see how it compared to a SynDaver™ skin tissue simulant. The puncture test adhered to the SynDaver™ specifications (Online supplementary Material-I Section 4), with oblique orientations (15 and 30°) introduced. The combined chamois leather and silicone were secured in the angle vice (Fig. 2A) and indented with a 1 mm diameter blunt iron rod at 15 mm s^−1^ till puncture (drop in force > 15%) was observed and the peak force was noted. The test location was moved (by at least 15 mm) and repeated twice (for a total of three punctures) for 0, 15 and 30° orientations each.

### Stud impact FE modelling methodology

**Figure 3:**
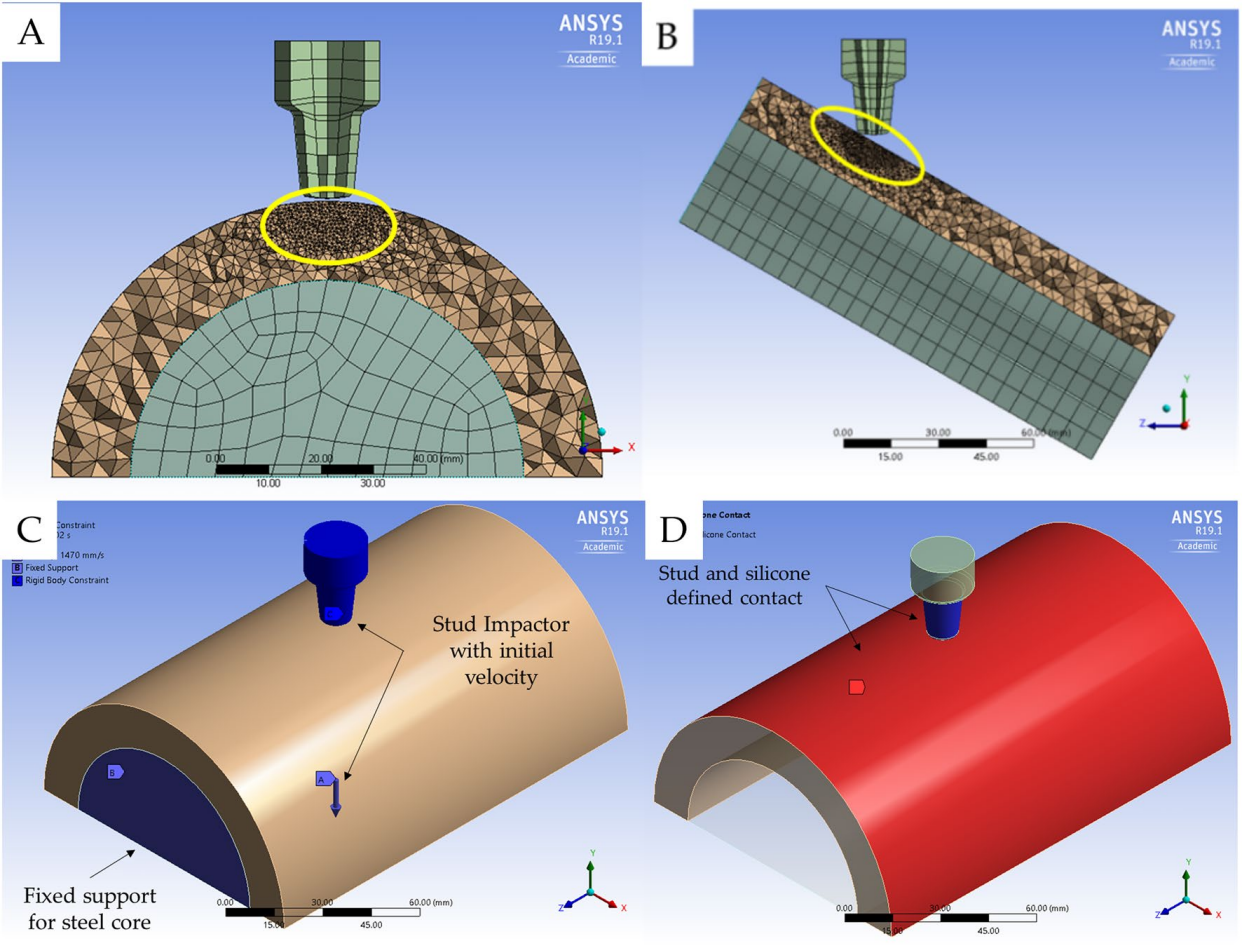
Model setup and mesh refinement applied around the impact site shown in (**A**) cross sectional layout and side view of (**A**) 0° and (**B**) side view of 30° orientation. The yellow ellipse highlights the region of refined mesh. (**C**) Boundary conditions applied for the testing setup and (**D**) contact region defined between the stud impactor (blue) and the silicone top surface (red).

The geometry of the surrogate and the stud impactor (matching the one used in the impact test and meeting the stud design requirements mentioned in Regulation-12 Schedule-2-Appendix E) were modelled in SolidWorks© (version 2018, Dassault Systems). The corresponding .sldprt file was imported into Ansys© Workbench Geometry using Design Modeller (Pennsylvania, USA). The geometric centre of the stud impactor was aligned to that of the surrogate in the widthwise (x-axis) and lengthwise (z-axis) directions. The lowest point of the impactor was placed 1 mm above (y-axis) the surrogate. For the 15° and 30° impact orientations, the surrogate geometry was rotated about the x-axis in Design Modeller to mimic the experimental setup (Fig. 3).

The impactor and hemicylinder were assigned rigid material models, with the properties of steel (*MAT_RIGID, Density-ρ = 7850 kg m^−3^, Youngs Modulus-E = 200 GPa and Poisson’s Ratio-ν = 0.3). The assigned density of the impactor was then artificially increased, as the entire drop carriage was not modelled, to match the mass of 3.65 kg in the experiment. The silicone and chamois leather were modelled as one material (combining the chamois leather and the silicone layer) and only damage to the top surface was simulated (i.e., the onset of skin damage). The material model assigned to the silicone was the same hyperelastic (5-parameter Mooney-Rivlin) model with rate dependency (2-term Prony series) as in Imam et al.^12^ (details reproduced in Table S1 of Online Supplementary material). In this work, damage criterion (*MAT_ADD_EROSION)^20^ was added to the material model. Maximum principal stress (SIGP1 in *MAT_ADD_EROSION card)^20^, based on potential shown during pilot simulations, was the variable used to trigger element erosion (i.e., the damage criterion).

Candidate maximum principal stress values for element deletion criteria were obtained by simulating the SynDaver™ puncture test on the silicone (See Fig. S2-C & Online Supplementary Material-1- Section 4-Puncture Test Experimental & Simulation Results). The applied force in the puncture test simulations ranged from 2 to 10 N, in steps of 2 N, corresponding to the range of four SynDaver™ skin tissue simulant puncture ratings. The maximum principal stress value from each of the five puncture test simulations (Online Supplementary Material-1 Table S6) was then independently assigned as the element deletion criteria in the model for the stud impact. Corresponding simulations were run at an impact energy of 4 J, at each of the three orientations, to determine which of the five candidates, maximum principal stress values best matched the experiment in terms of the impact force that initiated damage. The maximum principal stress for element deletion criteria for each orientation identified at 4 J was then used in corresponding simulations at 2 and 6 J and compared against the experimental data.

The stud impactor and hemicylindrical core were assigned the default hexahedral mesh (ELFORM = 1), giving a mean sizing of 6 mm. The silicone layer was assigned a tetrahedral mesh (ELFORM = 10) of size 3 mm. The mesh surrounding the target area of contact with the stud was further refined to 0.8 mm (mesh size setting) using a sphere of influence of diameter 20 mm (Fig. 3A), to improve the prediction of damage to the silicone (as determined from pilot simulations). A refined mesh was not applied to the entire silicone layer, as this was not needed for accurate damage prediction at the impact site. These settings gave a mesh with a mean quality of 0.85 ± 0.09 and a mean skewness of 0.218 ± 0.12 (detailed in Online Supplementary Material-1-Table S5). The boundary conditions in the model were set to replicate the experimental impact test setup. The hemicylindrical core of the shoulder surrogate was fully constrained. The stud impactor was assigned an initial velocity (*INITIAL_VELOCITY_RIGID_BODY) in the negative y-axis direction and constrained to only allow motion in the y-axis (Fig. 3C).

Pilot simulations with contact defined between the stud and silicone parts highlighted a drop in force once the elements in contact were deleted. Due to the compliant nature of the silicone, when an element was deleted, a shockwave propagation caused it to pass through the stud impactor^35^. As simulations with element deletion criteria for predicting damage are typically carried out between stiff materials, such as metal contacting metal or ceramic, they tend not to have high vibration amplitude. To overcome these issues, contact definition with the stud was limited to the top surface of the silicone. Contact between the stud outer surfaces and the top surface of the silicone (*AUTOMATIC_SURFACE_TO_SURFACE) was defined with a static and dynamic coefficient of friction of 0.3 (Fig. 3D), as per Imam et al.^12^. The contact force calculated between these surfaces was used to determine the value when the first element was deleted (i.e., force at silicone tear). Element deletion was identified through solution information text output at every timestep in Ansys﻿© WorkBench. This approach to contact modelling reduced the computational requirements but meant the model could only predict the onset of damage to the soft tissue simulant and not the extent (i.e., depth) of damage.

The impact velocities and corresponding defined durations of the simulations are in Online Supplementary Material-1 Table S4. The simulations were assigned a time step safety factor of 0.4 (Imam et al.^12^). The maximum vertical force (y-axis) before the silicone tore and the time to reach the tear was compared against the experimental results, i.e., the force and time corresponding to the onset of damage to the soft tissue simulant.

## Results

**Figure 4:**
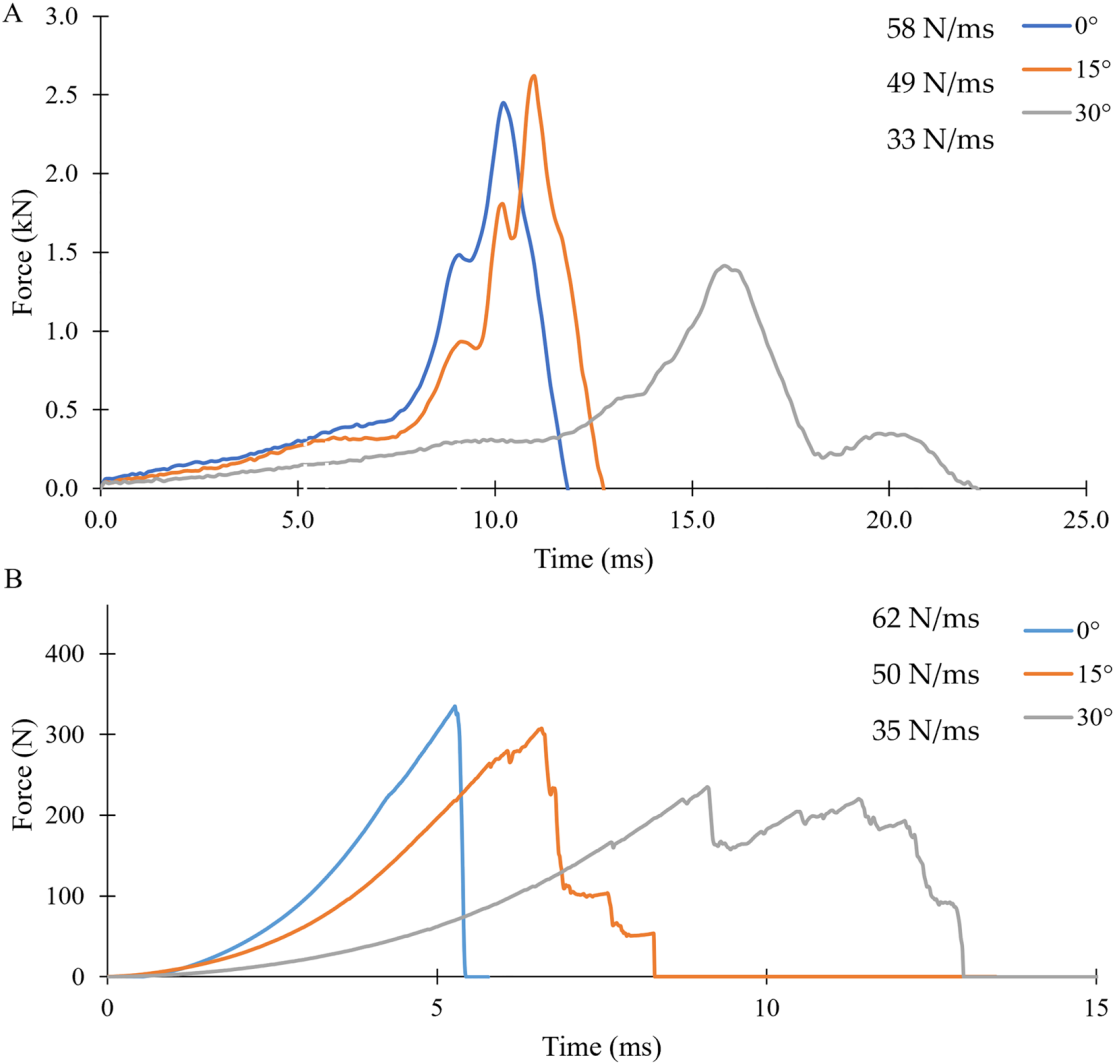
(**A**) Force trace and gradient of the impact at 4 J impact energy for experimental data at 0°, 15° and 30° orientation in comparison to the (**B**) Force trace from simulation with a linear loading gradient till failure with 16 MPa maximum principal stress element deletion criterion. Vertical dotted line indicates the time of tear break down on the force trace of the experimental impact along with HSV images is provided in Online supplementary Material 1-Figs. S4–6.

**Figure 5:**
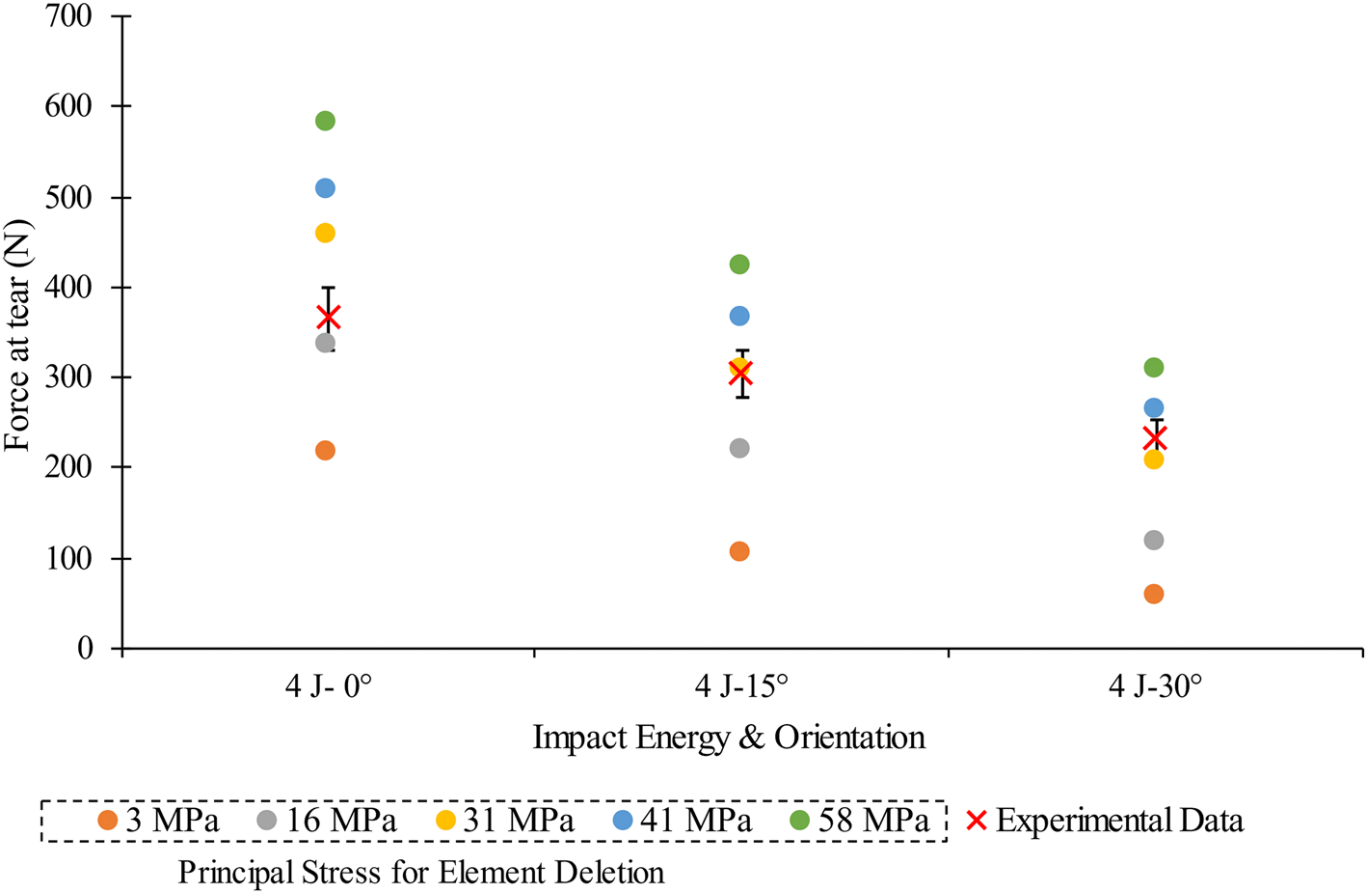
Peak force at tear values during a 4 J impact, at three orientations, using different maximum principal stress values compared against experimental data.

The experimental puncture force at 0° orientation for the chamois leather backed with silicone was around double that of the highest rated SynDaver™ skin tissue simulant (i.e., ~ 20 vs. 10 N). The experimental puncture tests showed that increasing the angular orientation between the indenter and the chamois leather reduced the force needed to cause a tear (details in Online Supplementary Materials -I-Fig. S1). The force to tear the silicone under a 4 J impact with the stud also decreased as the orientation angle increased (Fig. 4B). At 0° stud impact, the 16 MPa maximum principal stress element deletion criterion (identified in the 4 N applied indentation force simulation) gave the closest tear force to the experimental data (8% difference) (Fig. 5). For 15° and 30° impact angles, the 31 MPa element deletion criterion (6 N applied indentation force) gave the closest tear force to the experimental data (1 and 13%, respectively) (Fig. 5). Based on these results, the puncture force values from the experimental tests on the chamois leather (Fig. 2A and B) were not trialled in the model, as they would give maximum principal stress values for element deletion criteria that would cause an overprediction of tear force for 4 J impacts.

Peak forces predicted by the simulations with an impact energy of 2 J were lower than those from the corresponding experiment, as were the times to reach the peak force (Table 1). For 4 and 6 J impact energy, the force at which the silicone tore decreased with both the angle of impact and the impact energy. The time from when the stud struck the silicone until it tore increased with the impact angle. The simulations predicted a shorter time to tear the silicone than the experiment.

**Table 1:** Peak force and time to peak values for a 2 J stud impact simulation and simulation force and time to tear values for 4 and 6 J with percentage difference in comparison to experimental data.

Energy and angle	Peak force (N)			Time to peak (ms)		
	Experiment	FE	Difference (%)	Experiment	FE	Difference (%)
2J-0°	692	493	− 29	13.7	8.6	− 37
2J-15°	604	440	− 27	14.7	10.9	− 26
2J-30°	433	351	− 19	18.1	11.7	− 35

	Experiment		FE			
	Force at tear	Time to tear	Force at tear	Difference	Time to tear	Difference
	(N)	(ms)	(N)	(%)	(ms)	(%)
4J-0°	365	5.2	334	− 8	3.9	− 25
4J-15°	304	6.6	307	+ 1	5.4	− 17
4J-30°	232	9.1	263	+ 13	6.9	− 22
6J-0°	414	4.9	372	− 10	3.9	− 20
6J-15°	338	6.0	313	− 8	5.0	− 18
6J-30°	261	7.5	253	− 3	6.1	− 19

**Figure 6:**
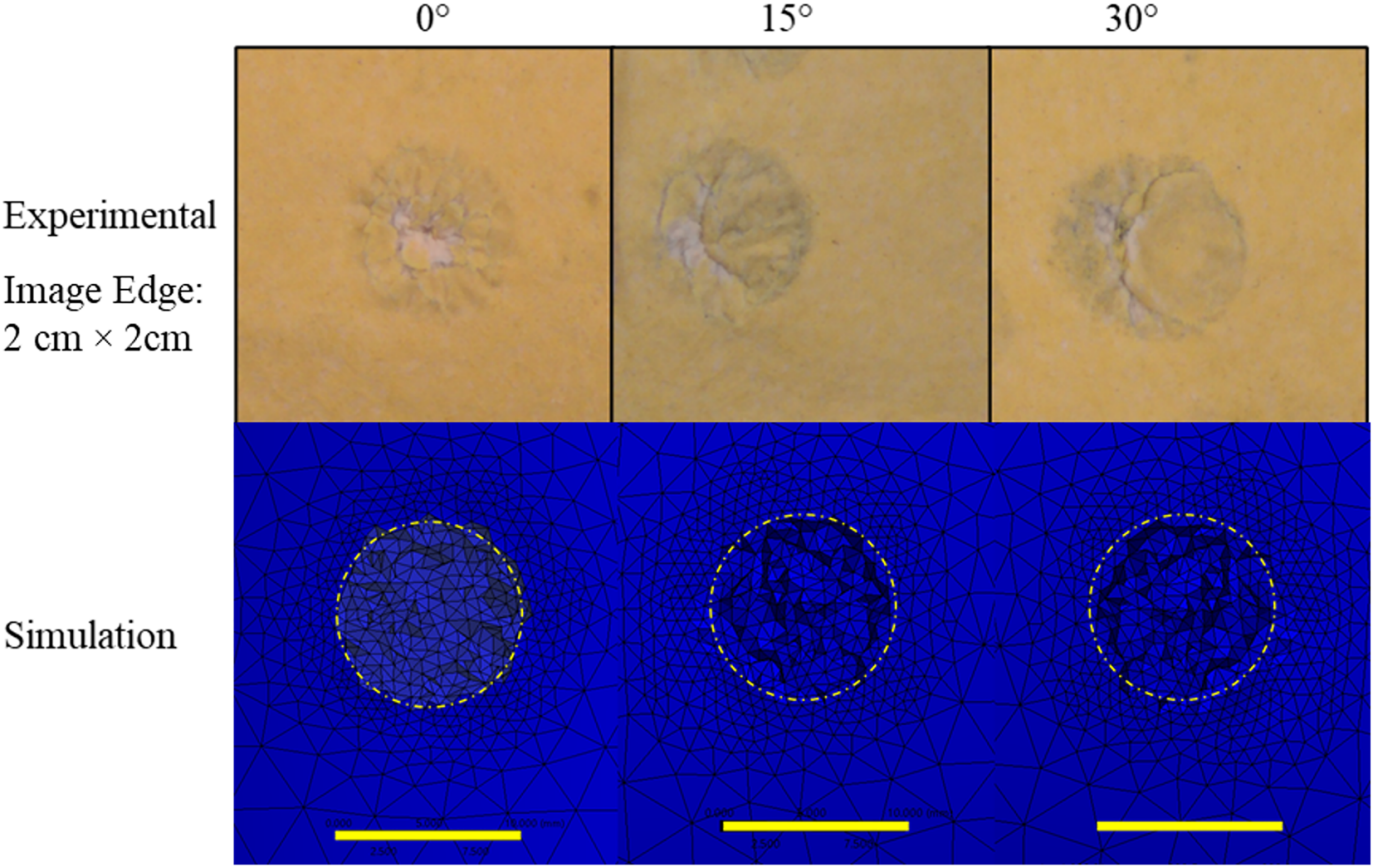
Nature of tear/damage occurring on the chamois leather following the impact at 4 J impact energy and different orientations after experimental testing in comparison to silicone damage during simulation of 4 J impact at 0°, 15° and 30°. 10 cm reference line and 10 mm diameter circle provided. (The difference in colouring is due to lighting orientations in Ansys©).

A visual comparison of damage to the chamois leather from stud impacts (photographs) and elements deleted is shown in Fig. 6. The damaged region was usually about the size of the impacting face of the stud. The nature of the damage was dependent on the orientation of the impact. For 0° impacts, the damage was circular with the tear in the chamois leather resembling a round hole. For angular impacts the damage was more elliptical, with the tear more pronounced on the side where the stud first struck.

## Discussion

The methods applied to model damage to the chamois leather used in the shoulder surrogate following a stud impact have been defined. A maximum principal stress of 16 MPa for the element deletion criteria provided the best agreement with the experimental stud impact test at 4 J-0° orientation in terms of the force required to tear the silicone. At the 15° and 30° orientations at 4 J, the simulation showed the best agreement with the impact experiment when a maximum principal stress of 31 MPa was used for the element deletion criteria. These results indicate that a different value should be used for each impact orientation to accurately predict chamois leather damage when using maximum principal stress as the failure criterion. This requirement to change the damage criterion value between impact scenarios is a limitation of the model that should be addressed in future work.

For the 2 J impact simulation, the peak force was lower than for the experiment. During experimental impacts at this energy, plastic deformation (i.e., permanent) of the chamois leather was noticed without an obvious tear. The simulation at 2 J did not predict a tear nor show any plastic deformation as a corresponding setting was not included in the model. The lower peak force for the simulation than for the experiment at 2 J impact energy may have been due to the plastic deformation of the soft tissue simulant during experimental testing. To overcome this issue, adding a setting to predict plastic damage (using a card like *MAT_ADD_GENERALIZED_DAMAGE in Ansys^20^) along with element deletion may improve force prediction under concentrated impacts where the silicone might deform permanently but not tear (i.e., those with low impact energy or less concentrated loads or both).

For the stud impact simulations at 4 and 6 J, the time to peak force and the time to tear the silicone was lower than for the experiment (ranging from 17 to 37%, Table 1). This underprediction was likely because of differences in the shape of the force vs. time curves between the experiment and simulations for distributed impact loads on the surrogate, as noted by Imam et al.^12^. As observed in these distributed impacts, the loading curve during the experiment with the stud had a shallower gradient than that of the simulation, meaning predicted force increased faster. As such, during stud impact testing, the model likely reaches higher stress values earlier than in the experiment, triggering element deletion too soon. Matching the impact loading response of the silicone in the model more closely to that of the experiment would likely reduce discrepancies in predicting the onset of damage.

In terms of maximum principal stress, candidate tear criteria for the chamois leather were obtained by simulating puncture tests used to define commercially available skin tissue simulants (SynDaver™ Tissue simulant). Replicating this test on the chamois leather in combination with the underlying silicone provided puncture forces exceeding those for the commercially available skin tissue simulant and far below those required to tear the silicone during the impact tests. Indeed, the puncture test used to determine candidate element deletion criteria differed substantially from the injury scenario simulated with a stud impact on the soft tissue simulant. In particular, the loading speed of the puncture test was much slower than the stud impact speed (15 vs. ~ 1500 mm s^−1^) and the diameter of the indenter was much smaller than that of the stud (1 vs. 18 mm). Obtaining the element deletion criteria with an indentation test that more closely mimics the injury scenario being simulated could lead to better results, potentially reducing the need to trail candidate failure criteria and then pick the one which works best. An option for further work could be to obtain the element failure criterion for an indentation test with the object used as the impactor in the impact scenario to be simulated. Such work could apply digital image correlation to quantify the accuracy of predicted strains on the surface of the soft tissue simulant during indentation and impact test simulations. Future work could also try other element deletion criteria, besides maximum principal stress, including applying more than one consecutively. Such an approach could remove, or lessen, the need to adjust the failure criterion to accurately model different impact scenarios.

For impact energies of 4 and 6 J, the model was within 15% of the peak force at tear values from the experiment (Table 1). During simulated impacts, the contact force acting between both the impactor and silicone and the hemicylindrical core and silicone were measured. When the stud struck the silicone, the corresponding contact force increased, as expected from the experimental data, until element deletion occurred. If some of the elements that made up the contacting surface remained, then a reduced force was detected (as noticed in angular impacts). Therefore, the stud-silicone contact force was only meaningful until the first element was deleted, signalling a silicone tear. Techniques such as re-meshing the body at every time step or defining contact between nodes and a surface (*AUTOMATIC_NODE_TO_SURFACE) or using a setting which redefines the contact surface at every time step (*ERODING_SURFACE_TO_SURFACE) could improve the stud-silicone contact force prediction throughout the impact, if this was of interest. Using such techniques would increase the simulation time.

An FE model with element deletion criteria, capable of predicting damage to a soft tissue simulant was developed. The simulation was run based on a range of puncture rating values and the element deletion criteria was identified for each orientation. Future work could incorporate padding into the model with a view to determining how it influences injury risk, i.e., whether it can prevent the onset of damage to the soft tissue simulant. Aside from stud impacts, the model could be developed to predict the onset of skin damage from other impactors, including those that represent anatomical structures, such as an elbow, knee, or head. Shoulder-to-shoulder, elbow-to-shoulder, or knee-to-shoulder impacts could be simulated to understand the force propagation and the risk of soft tissue injury. A model for artificial turf^36,37^ could be developed to simulate contact with skin to further knowledge on the types of skin injuries, particularly abrasions, that may occur. The techniques presented here could also be applied to soft tissue simulants for different skin tones, giving a more inclusive model for predicting soft tissue injury risk.

### Supplementary Information

Supplementary Information.

## Data Availability

The datasets generated during and/or analysed during the current study are available from the corresponding author on reasonable request.
