# Supplementary material for: A finite element model for predicting impact-induced damage to a skin simulant
Source: Sci Rep. 2024 Jun 4;14:12810. doi: 10.1038/s41598-024-60369-z (PMC11150437; doi:10.1038/s41598-024-60369-z)
Supplement: Supplementary file 1 — Supplementary Information. [file 41598_2024_60369_MOESM1_ESM.docx]

A finite element model for predicting impact-induced damage to a skin simulant.

Syed A Imam ^1,2^, Angus C Hughes^3^, Matt Carré^3^, Heather Driscoll^4^, Keith Winwood^2^, Prabhuraj Venkatraman^2^ and Tom Allen^2^

^1^ Canterbury Christ Church University, Canterbury, CT1 1QU, UK
^2^ Manchester Metropolitan University, Manchester M1 5GD, UK

^3^ Department of Mechanical Engineering, University of Sheffield, Sheffield S1 3JD, UK

^4^Advanced Manufacturing Research Centre (AMRC), Sheffield S60 5BL, UK

# Supplementary Information-I

## Material Properties of the silicone

Table 1: Summary of material data used for modelling the silicone used in the shoulder surrogate.

| Material Property / Setting | Value | Units |
| --- | --- | --- |
| Density | 1,072 | kg·m^-3^ |
| Poisson’s Ratio | 0.48 | - |
| Shear Modulus  (for frequency independent damping) | 47 | MPa |
| SIGF – limit setting for damping | 0.01 |  |
| Mooney-Rivlin Hyperelastic Coefficients | | |
| Incompressibility Parameter D_1_ | 0 | Pa^-1^ |
| Material Constant C_01_ | 1.01E+06 | Pa |
| Material Constant C_02_ | 2.47E+07 | Pa |
| Material Constant C_10_ | -9.30E+05 | Pa |
| Material Constant C_11_ | -3.60E+07 | Pa |
| Material Constant C_20_ | 1.37E+07 | Pa |
| Prony Series Terms | | |
| Relative Moduli (1) | 0.0116 |  |
| Relative Moduli (2) | 0.9347 |  |
| Relaxation Time (1) | 0.40463 | s |
| Relaxation Time (2) | 0.01005 | s |

## Compression testing to determine the consistency of the soft tissue samples

Five samples of the shoulder surrogate silicone moulded with chamois leather were made for impact testing (matching the design presented by Imam et al^7^). Each silicone sample was placed atop the steel core and subject to quasi-static compression testing before and after impact testing. This compression testing was to, i) measure the stiffness of each sample, ii) quantify inter-sample variance and iii) quantify any degradation from impact testing.

Each surrogate sample was compression tested using a uniaxial testing machine (Hounsfield HK10S) with a 1 kN load cell (0.5% or 5 N accuracy) and a 62 mm diameter flat indenter. Three different areas of the surrogate silicone sample (the surrogate was moved horizontally along the direction of its long axis to change the lengthwise position) were compressed to 20% strain (at a rate of 20 mm·min^-1^). The compression test was adapted from ISO2439-Method D (20% compression at a rate of 20 mm·min^-1^ and 30 s hold), but the hold after compression was not included as the intention of the test was to measure the instantaneous stiffness of the soft tissue simulant. The compression tests after impact testing for quantifying any degradation of the sample were at approximately the same locations as those from before impact testing. The force at 20% strain for the three compression tests taken on each sample before it was impact tested were compared across the six samples using Welch’s ANOVA (95% confidence level). The force at 20% compressive strain for all samples taken before and after impact testing were compared using Welch’s 2-Sample t-Test (95% confidence level).

No significant difference was found for force values at 20% compressive strain taken before impact testing across the six silicone samples (*p* = 0.108). The force values at 20% compression taken before and after impact testing were not significantly different (*p* > 0.05: range 0.078-0.476).

## Stud Impact Simulation Details

Table S2: Material properties of stud impactor and hemispherical core used during the stud impact modelling.

| Part | Density (kg·m^-3^) | Young's Modulus (GPa) | Poisson’s Ratio | Source |
| --- | --- | --- | --- | --- |
| Stud Impactor | 6.7E05 | 0.2 | 0.33 | Ansys© library – Aluminium with modified Density |
| Hemispherical Core | 7,850 | 200 | 0.30 | Ansys© library- Structural Steel |

Table S3 : Mesh details for different parts in the stud impact setup.

| Part | Elements | Nodes |
| --- | --- | --- |
| Stud Impactor | 154 | 216 |
| Silicone - 0° | 158,562 | 30,198 |
| Silicone - 15° | 155,164 | 29,660 |
| Silicone - 30° | 159,288 | 30,369 |
| Hemispherical Core | 1,898 | 2,430 |

Note: The number of elements and nodes for the silicone change with the angular orientation as the position and area under the sphere of influence change with orientation.

Table S4: Initial velocity of the stud impactor for the stud impact simulation.

| **Impact Energy (J)** | **Initial velocity (m/s)** | **End Time (ms)** |
| --- | --- | --- |
| 2 | 1.0 | 25 |
| 4 | 1.5 | 10 |
| 6 | 1.8 | 7.5 |

## Puncture Test- Experimental & Simulation Results

Experimental test as per the SynDaver test methodology showed that the puncture force was varied and reduced with an increase in the orientation (Figure S1).


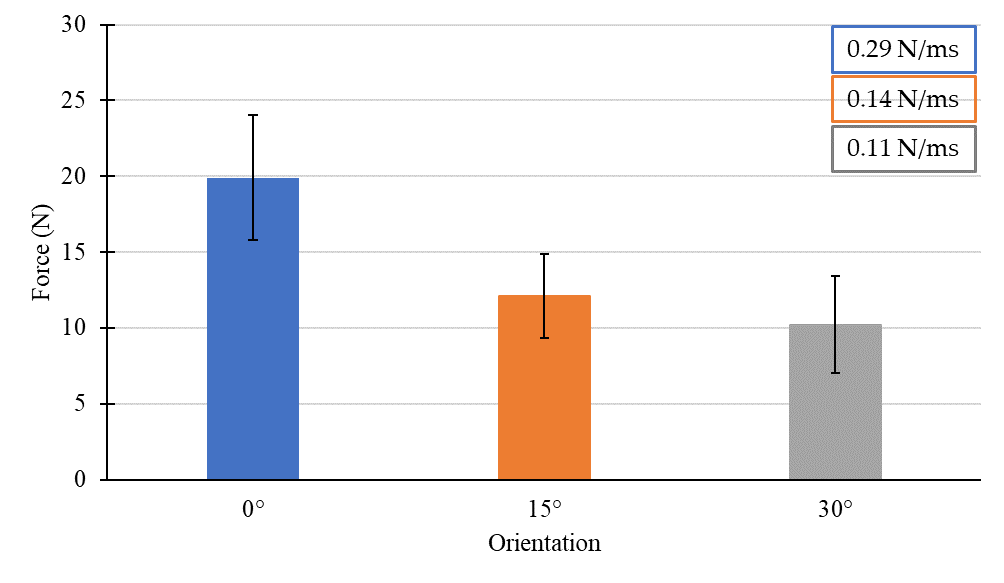


Figure S1: Puncture test results of the chamois leather moulded with silicone for each orientation along with their loading gradients. Error bars represent Standard Deviation.

The silicone layer was assigned a tetrahedral mesh of size 0.5 mm. A mesh refinement (of size 0.075 mm) was applied at the centre of the upper face of the silicone sample, surrounding the area of contact with the rod, using a sphere of influence (0.6 mm diameter). The rod was assigned default mesh settings resulting in an average edge length of 0.3 mm (Table S5).

Table S5: Mesh details for the simulant puncture test setup.

| **Part** | **Mesh Type** | **Elements** | **Nodes** |
| --- | --- | --- | --- |
| Rod | Hexahedral (ELFORM=1) | 574 | 840 |
| Silicone* | Tetrahedral (ELFORM=10) | 12,050 | 66,603 |

*The silicone and chamois leather were modelled as one material.

SynDaver™ defines the puncture test as a compression test with a 1 mm blunt Tungsten rod moving at 15 mm/s (Data Sheet shown in Figure 1). Geometry representing the puncture test, consisting of the rod (1 mm diameter with 0.5 mm filleted edges) and a cylinder (10 mm diameter and 3 mm thickness) for the silicone was modelled (Figure S3-A) in SolidWorks© (v2018, Dassault Systems). The corresponding .sldprt file was imported into Ansys© workbench geometry using Design Modeller. The rod was assigned a rigid material model (*MAT_RIGID), which was constrained to only allow for linear motion along the y-axis (i.e., vertical loading). The same material model (without damage criterion) as used in the impact simulations was assigned to the silicone, i.e., that of Imam et al.^12^ (5-parameter Mooney-Rivlin combined with a 2-term Prony series, Table S1). The mesh refinement on the model is shown in Figure S3-A. The underside and the vertical outer surface of the silicone geometry were fixed. Contact was defined between the silicone and rod with a static and dynamic coefficient of friction of 0.3 (*CONTACT_AUTOMATIC_SURFACE_TO_SURFACE). A constant force, ranging from 2 to 10 N (in steps of 2 N), was applied to the rod in the negative y-axis direction to compress it into the silicone (S2-B). The maximum principal stress occurring on the loaded surface of the silicone was noted (S2-C). The maximum principal stress noted for each applied load was then applied as the failure criteria in the stud impact simulation model to determine which value gave the closest match to the experimental impact test results in terms of the force required to tear the simulant.


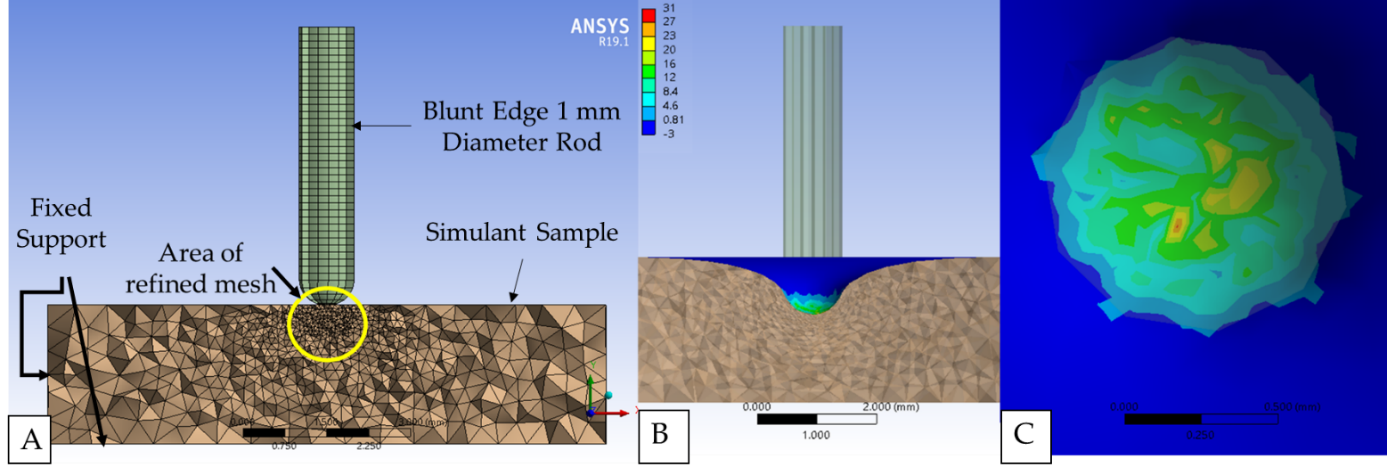


Figure S2: (A) Cross-sectional view of the puncture test showing the mesh refinement (B) Cross-sectional and (C) top-down view of contours of maximum principal stress (MPa) measured on the surface of the silicone layer under 6 N applied force during puncture test simulation.

For the indentation test simulations, the maximum principal stress values for the silicone increased with the force applied to the rod (Table S7).

Table S6: Maximum principal stresses measured off the surface of the silicone during puncture test simulation.

| Applied Force (N) | Maximum Principal Stress (MPa) |
| --- | --- |
| 2 | 3.1 |
| 4 | 15.9 |
| 6 | 31.0 |
| 8 | 41.3 |
| 10 | 57.8 |

## Experimental Stud Impact Force Trace Breakdown and Results

During the impact the stud can pierce through the silicone and strike the steel core of the shoulder surrogate, causing a sharp spike in force. For these impacts, the peak force does not coincide with the time at which the stud pierces the silicone. As such, simply comparing peak impact force values between the experiment and simulation is inappropriate for determining whether the model can accurately predict the scenarios that cause the silicone to tear or otherwise sustain damage. The general shape of the force traces for each orientation for impact energies of 4 and 6 J were similar (Figure S3-B & C). Therefore, only one force trace for the impact energy of 4 J at each orientation is presented, along with the corresponding HSV images, in Figures 4 to 6.


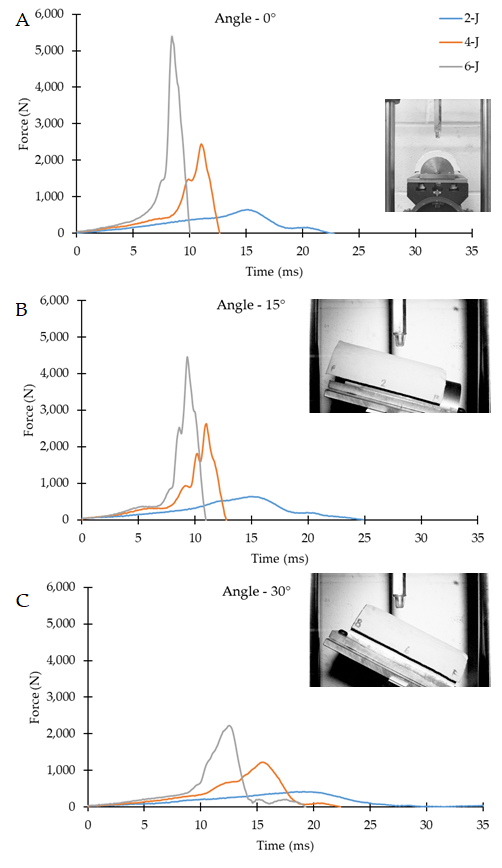


Figure S3: Force time trace for stud impact at (A) 0° (B) 15° and (C) 30° impact at 2, 4 and 6 J.

For a 4 J impact at 0°, the force increased gradually (at ~60 N/ms) from when the stud struck until the chamois leather tore (Point B on Figures S5-7), which then resulted in a slight reduction in the loading rate. The change in the force vs. time relationship after the silicone tore was less pronounced for the 0° orientation but can be clearly seen at 15° (Figure S6-Point B) and 30° (Figure S7-Point B) orientations. Following the tear, the impact force increased (between points B and C) as the silicone compressed until it split (Point C), with a corresponding drop in force. After the silicone split, the force increased rapidly to its peak value for the impact, suggesting that the stud struck the steel core of the shoulder surrogate (as can be observed in the HSV images). During impacts at 15° and 30°, the force trace had two local maxima after the silicone split.

During angular impacts, when the stud struck the steel core of the shoulder surrogate it deflected sideways. As the impactor straightened it regained contact with the steel core, causing an increase in vertical force. For the 15° orientation the second contact on the steel core gave the peak force for the impact (Figure S6 Point D), while for the 30° orientation, the peak force came from the first contact (Figure S7-Point D). For the 30° orientation, the effective thickness of silicone acting between the stud and steel core of the shoulder surrogate was higher, which explains the longer time to reach peak force (at ~16 ms). So, by the time the second contact on the steel core occurred, the force response was low as more impact energy had been transferred. Conversely, for the 15° impact, the sideways deflection of the stud impactor was visually less pronounced and hence the second contact on the steel core gave a higher force.

The graphs in Figures S5 to S7 indicated that while assessing the risk of injuries such as cuts and abrasions, taking the peak impact force would be ineffective. The peak impact force occurs when metal objects collide, causing a sharp peak. It is essential to understand the loading mechanics of the injury and the graphs help us identify some simple trends. As the impact angle increased, the loading rate decreased, from ~60 to 30 N/ms. The force at tear was higher at a higher loading rate but the time to tear was shorter.


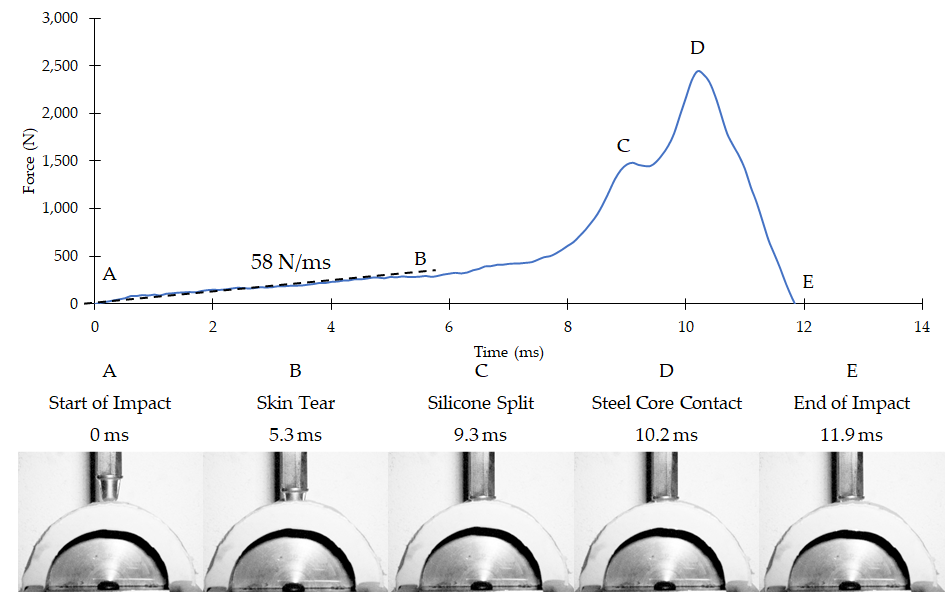


Figure S4: Breakdown of force trace of a 4 J impact at 0° along with the corresponding HSV images. The dashed line represents the loading trendline between A and B.


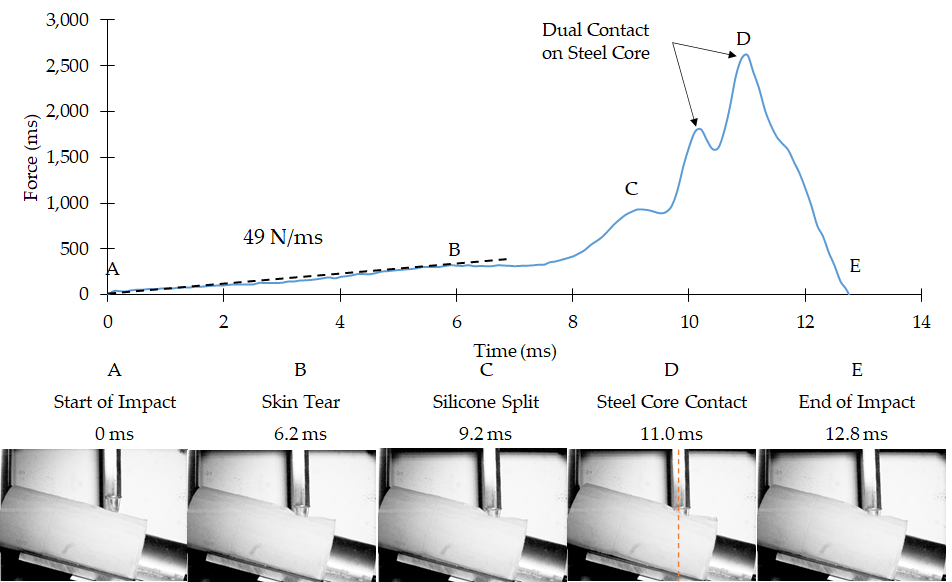


Figure S5: Breakdown of force vs. time trace of a 4 J impact at 15° along with the corresponding HSV images. The dashed line represents the loading trendline between A and B.


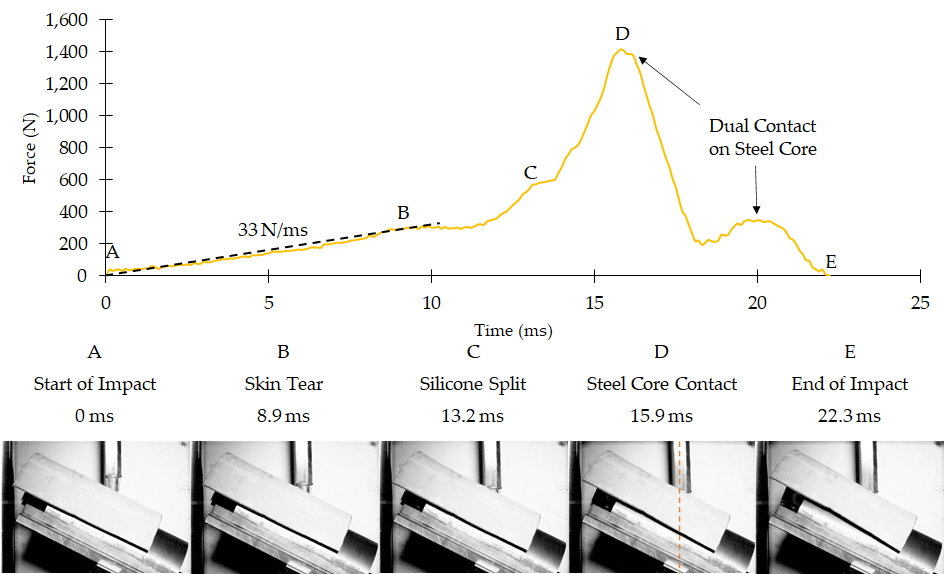


Figure S6: Breakdown of force vs. time trace of a 4 J impact at 30° along with the corresponding HSV images. The dashed line represents the loading trendline between A and B.
